# Steroid injection in chronic inflammatory vocal fold disorders, literature review

**DOI:** 10.1016/S1808-8694(15)30155-5

**Published:** 2015-10-18

**Authors:** Andrea Maria Campagnolo, Domingos Hiroshi Tsuji, Luís Ubirajara Sennes, Rui Imamura

**Affiliations:** a MD, Otorhinolaryngologist, PhD student in the Medical School at Universidade de São Paulo, professor of Otorhinolaryngology at Faculdade de Medicina de Campos; b Associate Professor at Hospital das Clínicas da USP, Assistant Physician at Hospital das Clínicas da Faculdade de Medicina da Universidade de São Paulo; c Associate Professor at Hospital das Clínicas da USP, Associate Professor of the Otorhinolaryngology Program at the Medical School at Universidade de São Paulo; d PhD in Otorhinolaryngology at Hospital das Clínicas da USP, Assistant Physician at Hospital das Clínicas da Universidade de São Paulo. Faculdade de Medicina da Universidade de São Paulo

**Keywords:** vocal fold scarring, steroids, steroid injection, inflammatory lesions of vocal fold, laryngeal disease

## Abstract

Steroids are potent inhibitors of inflammation and wound repair. Local administration of steroids directly into the larynx has been reported in many laryngeal diseases. **Aim**: The purpose of this study is to review related literature about the use of steroid injection in patients with benign, inflammatory and chronic vocal disease. **Methodology**: We performed an electronic survey in Medline database and selected clinical trials regarding steroid use in benign laryngeal diseases. **Results**: Steroids are indicated in these situations: 1) acute inflammatory diseases, mainly when edema compromises the airways; 2) auto- immune disease with laryngeal involvement; 3) laryngeal stenosis; 4) benign lesions of the vocal folds, e.g., nodules, polyps and Reinke”s edema, to reduce the inflammatory reactions before phonosurgery or in an attempt to avoid surgery; 5) In phonosurgery, aiming to reduce scarring. In this case, it could be used as a preventive measure in vocal fold scarring, or for scar treatment. **Conclusion**: Steroids may be considered an important therapeutic option in the management of many diseases, specially the inflammatory ones, associated with vocal changes.

## INTRODUCTION

**Figure 1:**
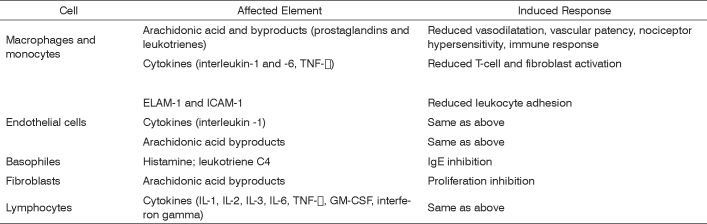
Sites and modes of action of glucocorticoids in inflammatory and immune responses.*

Steroids are molecules synthesized by our bodies to regulate a vast number of physiological, immune, and metabolic processes. Steroids are broadly used due to their anti-inflammatory and immunomodulation properties. They have a unique, essentially genomic (transcriptional) mode of action characterized by the activation (transactivation) or inhibition (transrepression) of numerous target genes. These molecules act upon a number of cells, involving innate (macrophages, granulocytes, mast cells) and adaptive (lymphocytes) immune responses, apart from other cells (fibroblasts, epithelial and endothelial cells). Steroid anti-inflammatory effectiveness is related to the inhibition of a number of cytokines, enzymes, and inflammation mediators and to the induction of cytokines and anti-inflammatory molecules such as lipocortin, responsible for inhibiting the release of vasoactive substances and chemotactic agents. Lipolytic and proteolytic enzymes are also reduced through lysosomal stabilization, as well as the overflow of leukocytes to sites of injury. Lymphocyte counts and degree of fibrosis are modified. All such steps will clearly affect the elements and stages of inflammatory response.^1^, ^2^ (Figure 1)

* Adapted from reference 2.

Steroids inhibit multiple sites of the immune system. They interfere with both humoral and cell immune response. Research findings suggest their effect in immune disease is more related to blocking inflammatory response than inhibiting immune response.^2^

**Figure 2:**
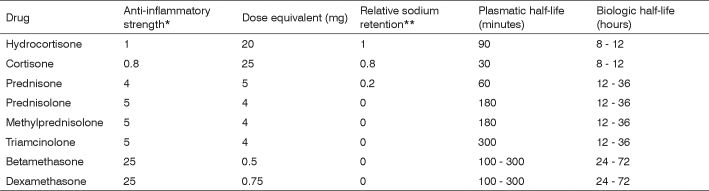
Glucocorticoid equivalence for systemic administration.

Synthetic steroidal drugs differ from each other in how strong their glucocorticoid is, and are thus dosed to provide for equal potency. They have different levels of mineralocorticoid activity and are categorized as a function of effect duration. (Figure 2)

* Glucocorticoid effect ** Mineralocorticoid effect Adapted from reference 3.

The glucocorticoid equivalence table considers systemic drug administration (intramuscular, intravenous, or subcutaneous). It is important to stress the difference between plasmatic and biologic half-life. The first refers to blood hormone levels, while the latter to the time for which the drug will be available in tissue; glucocorticoid tissue presence will determine the duration of its therapeutic effect. Such duration varies depending on the salt used in the formulation of the glucocorticoid. When prepared with salts that form freely soluble ester (mono and disodium phosphate, sodium succinate), glucocorticoids are promptly taken in. On the other hand, if the steroid is prepared with other salts that form less soluble byproducts (acetate, dipropionate, acetonide, diacetate, and hexacetonide) uptake is slower.^3^

Glucocorticoids are the most effective anti-inflammatory agent available, as they offer symptom alleviation in a series of clinical manifestations. They, however, pose a potential risk for adverse side effects in a number of organs depending on dosage and treatment duration. When used for short periods of time (up to two weeks), even at high dosages, the probability of adverse side effects occurring is low. In prolonged therapy severe side effects set in, thus reducing their effectiveness in addressing chronic diseases.^2^

Local steroid injections have been used as a means of rationalizing the use of this medication. This mode of administration allows for high local drug concentration with lower risk of systemic adverse side effect.

Steroids are mostly indicated to treat acute inflammatory diseases involving the larynx, mainly in situations where edema may compromise the airways. Some studies have shown steroids being used in various chronic diseases that introduce laryngeal lesions and voice alteration.

This study aims to perform a literature review on the use of steroid injections on the vocal folds of patients with benign, inflammatory, or chronic laryngeal disease.

## MATERIALS AND METHOD

A non-systematic search was performed on the MEDLINE database (from January of 1960 to November of 2006) to find medical papers reporting on the use of steroids in benign vocal fold disease.

The search for papers on Medline was done using keywords corticosteroids, steroid injection, vocal folds and laryngeal alone and in combination.

Other papers were also included besides the ones obtained from the above mentioned search.

## LITERATURE REVIEW

The use of steroid on managing laryngeal disease is well established, particularly in handling conditions such as epiglottitis, croup, and laryngeal edema.^4^, ^5^, ^6^

Steroids are potent inhibitors of inflammation and healing.

The most common administration modes are oral, intramuscular, and intravenous. Local steroid administration in the larynx has been reported for various laryngeal diseases.

Krespi^7^ reported on the use of steroid injections in the lesions of six patients with laryngeal sarcoidosis.

Patients with systemic lupus erythematosus and Wegener”s granulomatosis with laryngeal involvement present, in most of the cases, improvement in symptoms with steroid injections done directly on the laryngeal lesions.^8^, ^9^

Success has been achieved in the treatment of patients with laryngeal stenosis with injections of triamcinolone on the site of stenosis.^10^, ^11^

Some authors have reported on the use of steroid injections on the vocal folds to treat local inflammatory benign diseases such as nodules, polyps, and Reinke”s edema, in an attempt to avoid surgery.

In 1967, Yanagihara^12^ reported good results after injecting dexamethasone on vocal folds assisted by indirect laryngoscopy in patients with nodules. This technique is however quite inaccurate, thus hampering its reproducibility. Tateya et al.^13^ developed a technique using a fibroscope and a curved needle introduced orally to inject triamcinolone under local anesthesia on vocal fold nodules. Twenty-three of the 27 patients enrolled in this study had nodules associated with vocal abuse and occupational stress (20 were teachers). Endoscopic findings showed that vocal fold nodules disappeared in 17 of the 27 subjects and were reduced in size in 10 patients after injection. Maximum phonation time was of 10.9s before the procedure and moved up to 13.9s afterwards, showing a significant increase (p<0.05). Remission or improvements in dysphonia were seen in 96% of the cases. There was no recurrence of adverse side effects such as reduction on vocal fold size or muscle atrophy.

These same authors^14^ conducted a study using this same technique to inject triamcinolone on Reinke”s space of the vocal folds of 44 patients with moderate Reinke”s edema. Remission and improvements were observed in almost all patients as far as dysphonia is concerned (assessed by patients) and in vocal fold endoscopic findings. The mean maximum phonation time increased significantly from 9s to 11.4s after the procedure (p<0.01). In female patients, pitch also increased significantly from 168Hz to 181Hz (p<0.05) after steroid injection.

Surgical technique and instrumentation used to remove benign vocal fold lesions and yet preserve maximum amounts of normal adjacent tissue have improved along the years. Understanding the body-cover vocal fold model is fundamental for any surgical approach. A mucosal wave is generated as the mucosal lining slides over the vocal ligament and the vocal muscle. This cover can be divided into layers with different mechanical properties, and may be differentiated by the concentration of collagen and elastin fibers. Most benign lesions take place in the superficial layer of the lamina propria, and surgical approaches should ideally be confined to this layer. Violation of the deeper layers of the lamina propria and the vocal ligament is associated with scar tissue formation. Such scar leads to the fixation of the mucosal cover to deep tissues, thus hampering the formation of the mucosal wave and causing dysphonia. Vocal fold scars are the most common cause of post-surgery dysphonia (35%).^15^

Steroids have been clinically used in vocal fold surgery to reduce scar tissue formation; they may also be used preventively or therapeutically against scar tissue.

Bouchayer and Cornut16 reported on the use of hydrocortisone injections on the vocal folds at the end of microsurgery to remove benign lesions such as nodules, manly when sings of inflammation, cysts, sulcus, and bridges are present. When treating iatrogenic scars, hydrocortisone is injected on the vocal fold, an incision is made in the superior surface of the vocal fold, and a microflap is raised separating it from the vocal ligament, without however removing any type of tissue. The authors reported improvements on vocal fold malleability, glottic closure, and voice quality. Courey et al.^17^ have also described the use of steroids under the microflap after benign lesion removal so as to reduce scar tissue formation consequent to the microflap.

Mortensen and Woo^18^ injected methylprednisolone on the vocal folds of 12 patients with post-surgery iatrogenic fibrosis assisted by indirect laryngoscopy under local anesthesia. Significant improvements were observed on voice quality, as measured by the GRABS scale (p<0.01). In stroboscopic examination improvements were seen on vibration amplitude (p<0.05) and mucosal wave (p<0.05). This study also looked at 18 patients with vocal folder nodules or polyps and 4 patients with sarcoidosis or granuloma. Eleven of the 18 patients with nodules or polyps had significant improvement and did not require surgery. One patient with sarcoidosis was able to avoid repeated general anesthesia procedures with steroid injection. Two patients with contact granuloma improved with steroid injections and did not require surgery. One patient required surgery and pathology test results showed vocal fold tuberculosis. Twenty-eight (82%) of the 34 patients improved their statuses. The authors reported that on-lesion steroid injections have three main indications:

1) reduce granulation tissue and promote primary healing;
2) reduce hypertrophic scar formation; and
3) reduce inflammation to avoid surgery.

The use of steroid injection to prevent vocal fold fibrosis is based only on theory, as there are no clinical or histological studies presenting consistent findings on the effect of steroids in vocal fold healing.

The only published study that histologically and functionally analyzes the effect of vocal fold steroid injection was produced by Coleman et al.^19^ in 1999. A microflap lateral to the vocal folds of 15 dogs was raised and triamcinolone was injected on one of the vocal folds; the other was left for control purposes. After 2, 4 and 6 weeks respectively groups of five dogs were slaughtered and vocal fold histological studies were done to analyze inflammatory infiltrate and neovascularization. Paired tests showed increased presence of inflammatory infiltrate around the microflap of vocal folds treated with steroids for 2, 4 and 6 weeks (p<0.2). Neovascularization in vocal folds treated with steroids was reduced in 2 weeks (p<0.005), but greater in 4 and 6 weeks (p<0.005). A time-dependent normal distribution curve was produced based on histological data to better characterize the effect of steroids upon the healing process. Steroid-treated tissues had responses delayed by 12 days for inflammatory infiltrate and 21 for vasculogenesis. Videostroboscopy was done preoperatively and the day the dogs were slaughtered to allow for a functional qualitative and quantitative analysis. No significant differences were found in appearance, amplitude, mucosal wave, or malleability between the two vocal folds. In this study, although the healing process was delayed by steroids, the parameters assessed during videostroboscopy did not show differences between treated and control vocal folds after 2, 4 and 6 weeks.

Scars are generally related to fibrous tissue. Studies have shown that increases in collagen - especially type 1 collagen - promote fibrosis, and that collagen content has direct impact on scar strength.^20^ In the case of vocal fold fibrosis, dense collagen deposits have also been found in the injured lamina propria of animal model vocal folds^21^, ^22^, ^23^. However, recent research suggests that other components of the extracellular matrix such as hyaluronic acid, fibronectin, and decorin may also affect fibrous tissue. Although collagen is the main molecule, it is not the only one to determine tissue viscoelastic properties.^24^

A study on the role of monocytes/macrophages in healing, subcutaneous deposit hydrocortisone at 0.6mg/g of bodily weight was given to pigs to induce long term monocytopenia. Macrophage level in the wound was reduced to 1/3 in relation to control subjects. Fibroblasts would normally appear within three days, but were not seen before 5 days and their proliferation was reduced on the healing site.^25^

Injections of deposit steroids such as triamcinolone have been used in hypertrophic scars and cheloid. Carroll et al.^26^ showed in vitro that collagen and cell proliferation reduction promoted by triamcinolone is mediated by alterations in cytokine secretion.

A study is currently being conducted in animal models at our service to histologically assess whether steroids can be used in the formation stages of the extracellular matrix components as an alternative to reduce vocal fold mucosal stiffness introduced by postoperative healing. Twelve rabbits were used in this study. All subjects got dexamethasone injections through a lateral microflap raised in one of their vocal folds, while the other vocal fold was left with the microflap untreated for control purposes. Histology tests were done to assess inflammatory infiltrate and amount of collagen after three and seven days, during acute inflammatory response. In terms of inflammatory infiltrate, there was no difference in the number of cells around the microflap. Amounts of collagen, however, were significantly lower in the steroid-treated vocal fold after three days, and this difference was sustained after seven days.^27^

## DISCUSSION

Steroids are broadly employed in various laryngeal diseases, mainly because of their powerful anti-inflammatory properties. Alongside benefits are, however, various potential adverse side effects that may involve a series of tissues depending on dosage and treatment duration. Any agent can be administered in systemic acute conditions, as undesired side effects are not observed.

When the therapeutic goal involves reaching higher concentrations in one single target - e.g.: larynx - local steroid injection is effective and introduces fewer systemic side effects, as long as dosage is strictly observed and treatment is short term. Longer half-life steroids such as triamcinolone, methylprednisolone acetate, and dexamethasone are preferably used in local injections. Triamcinolone is broadly used in joint injections in the orthopedic practice^28^, ^29^, with confirmed effectiveness and safety. Bouchayer and Cornut16 used vocal fold hydrocortisone injections to reduce present signs of inflammation, but probably due to shorter medication half-life the impact on healing was limited. In theory, longer half-life steroids do a better job of preventing fibrous tissue formation.

The main adverse effect of this treatment mode is muscle and gland atrophy. None of the papers referred to in this study reported such event. However, Mortensen and Woo18 suggest patients with fibrosis should have intervals of 6 to 12 weeks between injections and those suffering from inflammatory lesions should either have one single injection or more in longer intervals.

Many autoimmune diseases such as systemic lupus erythematosus, Wegener”s granulomatosis, and sarcoidosis may present laryngeal lesions that cause dysphonia and respiratory involvement. Most symptoms are resolved with steroid therapy, usually employed for longer periods of time. Immunosuppressant drugs and chemotherapy medication (azathioprine, cyclophosphamide, and methotrexate) are also associated to reduce or stop steroid administration. However, patients with severe laryngeal involvement have improved when taking local steroid injections, possibly an alternative with fewer adverse side effects.

Healing modulation is the biggest factor in the success of stenosis surgical treatments. Steroids were tested with this purpose, but their use remains controversial^30^. On the one hand, they act on collagen formation and degradation, reducing granulation tissue formation, as observed by Gnanapragasam^10^ and Rosen and Vered^11^. On the other hand, they interfere with the migration of epithelial cells and on the healing of developing ulcers, which may get infected and increase the chances of restenosis. Local steroid injection may also produce cartilage resorption.31 Mitomycin-C has shown more promising results than steroids to treat laryngeal stenosis.^32^

Acquired benign laryngeal lesions affecting the vocal folds are the consequence of inflammatory processes that stem basically from three factors: vocal fold abuse or poor use of the voice (trauma), irritative processes (smoking, pharyngeal-laryngeal reflux, pollution etc), and individual predisposing factors.^33^

Vocal fold nodule disease is characterized by the distension of capillaries and venules, mild perivascular hemorrhage, tissue fibrin migration, diffuse edema, and fibrosis. Polyps, Reinke”s edema, and contact granuloma also have inflammatory traits. Thus, local steroid injections are a rational and particularly effective means to treat these lesions in their early stages, before fibrous tissue has developed.^13^, ^33^

Vocal fold fibrosis is caused by injury and inflammation. Inadequate surgical technique can easily introduce fibrous scars on the vocal folds. These scars disorganize the vocal fold mucosal lamina propria and change the biomechanical properties of the vocal folds, as mucosal viscoelastic strength is increased. This, in turn, reduces vocal fold vibration and causes dysphonia, a condition difficult to be treated.^24^

No treatment method can yet be considered effective in resolving vocal fold fibrosis.^24^ Steroid injections are among the most broadly employed treatments and have been associated with reduced rigidity, improved glottal closure, and good resulting voice quality, as observed by Bouchayer and Cornut^16^ and Mortensen and Woo^18^. Nonetheless, some degree of fibrosis always remains in place. Many other substances have been attempted, such as bovine collagen^34^, autologous collagen^35^, and autologous fat^36^ to enhance vocal fold flexibility. However, none of these materials is able to restore the changes introduced in the lamina propria by fibrosis and repair viscoelasticity to levels identical to those found in normal vocal folds.

As the treatment options for vocal fold fibrosis are limited, studies have been conducted to prevent fibrosis formation. Many surgeons are using steroid injections on surgical wounds to enhance injury healing. Steroids affect collagen synthesis and maturation, change wound tensile strength, inhibit fibroblast function, and suppress defense cell antibacterial and phagocytic properties, resulting in modified patterns and delayed wound healing^37^. The effect is more visible within the first four days of treatment. Aside from inhibiting normal infection resistance, after four days steroids have almost imperceptible effect^38^. According to Branski et al.^39^, the acute stage of the healing process is critical. Therapeutic interventions introduced at this time may reduce vocal fold scar stiffness, as inflammatory cells and fibroblasts start synthesizing extracellular matrix components two or three days after injury. If fibrosis prevention is the goal, the right time to use long half-life steroids is at the onset of the inflammatory process, i.e., at the end of surgery.

The alterations introduced by steroids in collagen synthesis were reported by Campagnolo e Tsuji^27^, but they are associated only with the acute inflammatory stages. Coleman et al.^19^ followed their patients for 6 weeks and found no difference in vocal fold vibration under functional stroboscopic assessment. Histological examination did not consider matrix components (collagen, elastin etc) and consequently their association with vocal fold mucosa vibration; they looked only at inflammatory infiltrate and neovascularization. In spite of the proven effect of steroids in healing, more studies are required mainly to assess whether collagen changes persist in the long run.

Reports on the use of mitomycin-C have not shown benefit in preventing fibrosis.^40^ Encouraging results were recently reported from liver cell growth factor injections, as a potent anti-fibrotic agent.^41^ Ideal prevention and treatment modes for vocal fold fibrosis are yet to be established.

## CONCLUSION

Systemic steroids are recommended to treat acute laryngeal inflammation. In chronic inflammatory disease, steroids may be used systemically or injected locally on the lesion in more severe cases.

Local steroid injections are advantageous as they allow higher drug concentrations on the target with reduced potential for adverse systemic side effects. They may be indicated to 1) reduce edema, benign mucosal lesion, and granuloma size before surgery or as an attempt to avoid it, and 2) minimize post-surgery iatrogenic scar. Although many authors reported on local steroid injection at the end of surgery to prevent scar formation, such application remains controversial.

Steroids may be considered as an important treatment option for various diseases, mainly inflammatory ones that adversely affect the vocal folds.
